# Complete chloroplast genome sequences of the ornamental plant *Prunus cistena* and comparative and phylogenetic analyses with its closely related species

**DOI:** 10.1186/s12864-023-09838-9

**Published:** 2023-12-05

**Authors:** Lijuan Feng, Guopeng Zhao, Mengmeng An, Chuanzeng Wang, Yanlei Yin

**Affiliations:** 1 Shandong Institute of Pomology, Taian, 271000 Shandong China; 2 Yantai Testing Center for Food and Drug, Yantai, 264005 Shandong China; 3 Zibo Academy of Agricultural Sciences, Zibo, 255000 Shandong China; 4 Shandong Academy of Agricultural Sciences, Jinan, 250100 Shandong China

**Keywords:** *Prunus cistena*, Chloroplast genome, Genetic diversity, Phylogenetic analysis

## Abstract

**Background:**

*Prunus cistena* is an excellent color leaf configuration tree for urban landscaping in the world, which has purplish red leaves, light pink flowers, plant shape and high ornamental value. Genomic resources for *P. cistena* are scarce, and a clear phylogenetic and evolutionary history for this species has yet to be elucidated. Here, we sequenced and analyzed the complete chloroplast genome of *P. cistena* and compared it with related species of the genus *Prunus* based on the chloroplast genome.

**Results:**

The complete chloroplast genome of *P. cistena* is a 157,935 bp long typical tetrad structure, with an overall GC content of 36.72% and higher GC content in the in the inverted repeats (IR) regions than in the large single-copy (LSC) and small single-copy (SSC) regions. It contains 130 genes, including 85 protein-coding genes, 37 tRNA genes, and 8 rRNA genes. The *ycf3* and *clpP* genes have two introns, with the longest intron in the trnK-UUU gene in the LSC region. Moreover, the genome has a total of 253SSRs, with the mononucleotide SSRs being the most abundant. The chloroplast sequences and gene arrangements of *P. cistena* are highly conserved, with the overall structure and gene order similar to other *Prunus* species. The *atpE*, *ccsA*, *petA*, *rps8*, and *matK* genes have undergone significant positive selection in *Prunus* species. *P. cistena* has a close evolutionary relationship with *P. jamasakura*. The coding and IR regions are more conserved than the noncoding regions, and the chloroplast DNA sequences are highly conserved throughout the genus *Prunus*.

**Conclusions:**

The current genomic datasets provide valuable information for further species identification, evolution, and phylogenetic research of the genus *Prunus*.

**Supplementary Information:**

The online version contains supplementary material available at 10.1186/s12864-023-09838-9.

## Background

*Prunus cistena* is an ornamental tree belonging to the genus *Prunus* within the Rosaceae family and is popularly cultivated in China. The genus *Prunus* consists of over 250 species, appreciated worldwide for their beautiful flowers and leaves, making them of high ornamental and economic value [1–3]. Due to their potential for development and application, research on this economically important group has become increasingly extensive [4, 5]. *P. cistena* displays purplish red or dark purplish red leaves with light pink flowers. Its distinctive plant shape and high ornamental value make it an excellent choice for urban landscaping.

Chloroplasts are unique organelles in green plants and play a crucial role in photosynthesis, amino acid synthesis, and carbon sequestration [5–7]. The chloroplast genomes of higher plants typically consist of a quadripartite structure, including two inverted repeats (IRs) of 20–28 kb, separated by a large single-copy (LSC) region of 80–90 kb and a small single-copy (SSC) region of 16–27 kb. These genomes exhibit highly conserved gene content and order [8–10]. Notably, the chloroplast genome of *P. cisterna*, like others in the genus, exhibits characteristics of haploid inheritance, a relatively small genome, a slow mutation rate, and sufficient polymorphism, making it an ideal model for genomic evolution and the development of molecular markers for resolving phylogenetic relationships [11–13].

Recent advancements in high-throughput sequencing technologies have made the assembly of chloroplast genomes more convenient and cost-effective for conventional species. Numerous studies have highlighted the chloroplast genome variations' effectiveness in identifying and resolving phylogenetic relationships at different levels [14–17]. While the chloroplast genomes of several *Prunus* species, such as *P. pseudocerasus* [2], *P. campanulata* [3], and *P. phaeosticta* [5], have been sequenced, complete studies on the chloroplast genome of *P. cistena* are still lacking, limiting the exploration of its genetic information and comprehensive analysis of interspecific relationships within the genus *Prunus*.

The molecular structures and phylogenetic relationships of 20 *Prunus* subgenus *Cerasus* species were comparatively analyzed based on complete chloroplast genomes [4]. The nuclear and chloroplast SSR markers were used to distinguish different genetic lineages of cultivated almond and characterize an extensive gene pool for genetic improvement [18]. However, the chloroplast genomes of *P. cistena* have yet to be reported, leading to limitations in mining its genetic information and comprehensive analysis of the interspecific relationships of *P. cistena* and other species in the genus *Prunus*.

Therefore, this study presents the first report on the complete chloroplast genome sequences of *P. cistena*. We investigated the phylogenetic tree of *P. cistena* and other species within the Rosaceae family and conducted a comparative genomic analysis among six species of the genus *Prunus*. Our findings lay a foundation for future genomic research on phylogenetic relationships and evolutionary patterns within the genus *Prunus*.

## Results

### Characteristics of *P. cistena* cp genomes

**Fig. 1:**
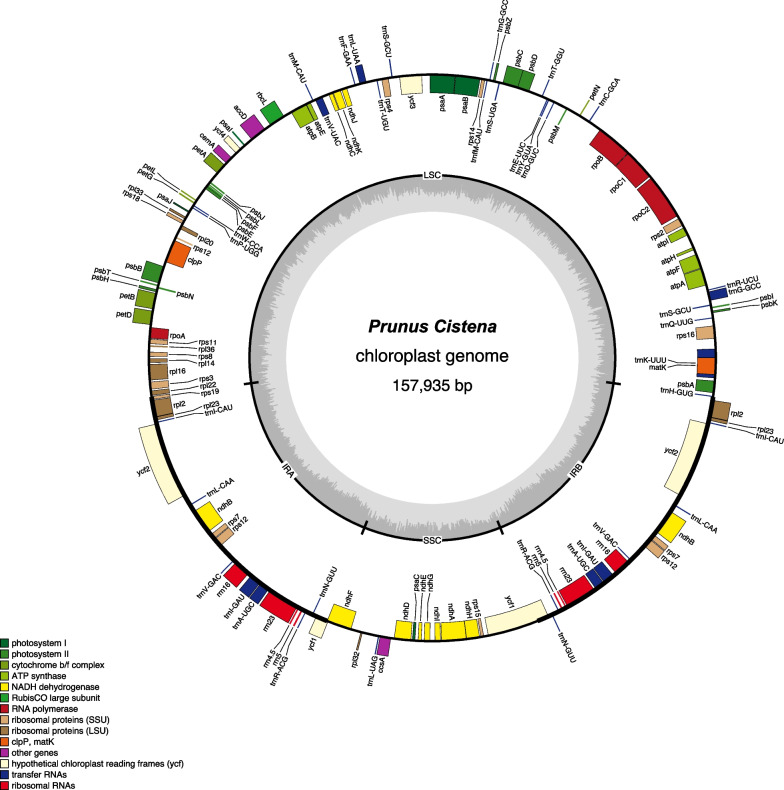
Chloroplast genome maps of *Prunus Cistena*. Genes belonging to functional group are color-coded. The positive coding gene is located on the outside of the circle, and the reverse coding gene is located on the inside of the circle. The grey circle inside circle represents the GC content

The complete cp genome of *P. cistena* is a typical 157,935 bp long circular double-stranded DNA structure with a quadripartite structure. It comprises one LSC region of 85,947 bp, one SSC region of 19,116 bp, and a pair of IR regions of 26,436 bp each (Fig. 1). The overall GC content of the genome is approximately 36.72%, with the IR regions exhibiting a higher GC content of 42.53% compared to the GC content of LSC (34.59%) and SSC (30.22%). This GC content distribution pattern is consistent with that observed in other plants [19–21].

The cp genome of *P. cistena* contains 130 genes, including 85 protein-coding genes (PCGs), 37 tRNA genes, and 8 rRNA genes (Table 1). Among the 85 PCGs, nine are for large subunits of the ribosome, 12 for small subunits of the ribosome, four for RNA polymerases, 20 for the photosystem, and six for ATP synthases. Additionally, the IR regions contain 20 duplicated genes, including seven PCGs, nine tRNA genes, and four rRNA genes (Table 1). Among the 23 intron-containing genes, 21 include a single intron, and two genes (*ycf3*, *clpP*) have two introns (Table S1). In comparison with other introns, the *trnK-UUU* gene in the LSC region has the longest intron of 2547 bp, and the *trnL-UAA* gene has the shortest intron of only 514 bp. The *rps12* gene is a trans-spliced gene with a single 5′-end at the LSC region and repeated 3′-end exons in the IRs (Fig. 1 and Table S1).

**Table 1:** Annotated genes in the *Prunus Cistena* CP genomes

Category	Gene group	Gene name
Photosynthesis	Subunits of photosystem I	psaA, psaB, psaC, psaI, psaJ
	Subunits of photosystem II	psbA, psbB, psbC, psbD, psbE, psbF, psbH, psbI, psbJ, psbK, psbL, psbM, psbN, psbT, psbZ
	Subunits of NADH dehydrogenase	ndhA^a^, ndhB^a^(2), ndhC, ndhD, ndhE, ndhF, ndhG, ndhH, ndhI, ndhJ, ndhK
	Subunits of cytochrome b/f complex	petA, petB^a^, petD^a^, petG, petL, petN
	Subunits of ATP synthase	atpA, atpB, atpE, atpF^a^, atpH, atpI
	Large subunit of rubisco	rbcL
Self-replication	Proteins of large ribosomal subunit	rpl14, rpl16^a^, rpl2^a^(2), rpl20, rpl22, rpl23(2), rpl32, rpl33, rpl36
	Proteins of small ribosomal subunit	rps11, rps12^b^(2), rps14, rps15, rps16^a^, rps18, rps19, rps2, rps3, rps4, rps7(2), rps8
	Subunits of RNA polymerase	rpoA, rpoB, rpoC1^a^, rpoC2
	Ribosomal RNAs	rrn16(2), rrn23(2), rrn4.5(2), rrn5(2)
	Transfer RNAs	trnA-UGC^a^(2), trnC-GCA, trnD-GUC, trnE-UUC, trnF-GAA, trnG-GCC, trnG-GCC^a^, trnH-GUG, trnI-CAU(2), trnI-GAU^a^(2), trnK-UUU^a^, trnL-CAA(2), trnL-UAA^a^, trnL-UAG, trnM-CAU, trnN-GUU(2), trnP-UGG, trnQ-UUG, trnR-ACG(2), trnR-UCU, trnS-GCU(2), trnS-UGA, trnT-GGU, trnT-UGU, trnV-GAC(2), trnV-UAC^a^, trnW-CCA, trnY-GUA, trnfM-CAU
Other genes	Maturase	matK
	Protease	clpP^b^
	Envelope membrane protein	cemA
	Acetyl-CoA carboxylase	accD
	c-type cytochrome synthesis gene	ccsA
Genes of unknown function	Conserved hypothetical chloroplast ORF	ycf1(2), ycf2(2), ycf3^b^, ycf4

^a^contains one intron; ^b^contains two introns; (2): gene with a copy number greater than 1, the number of copies in parentheses

**Fig. 2:**
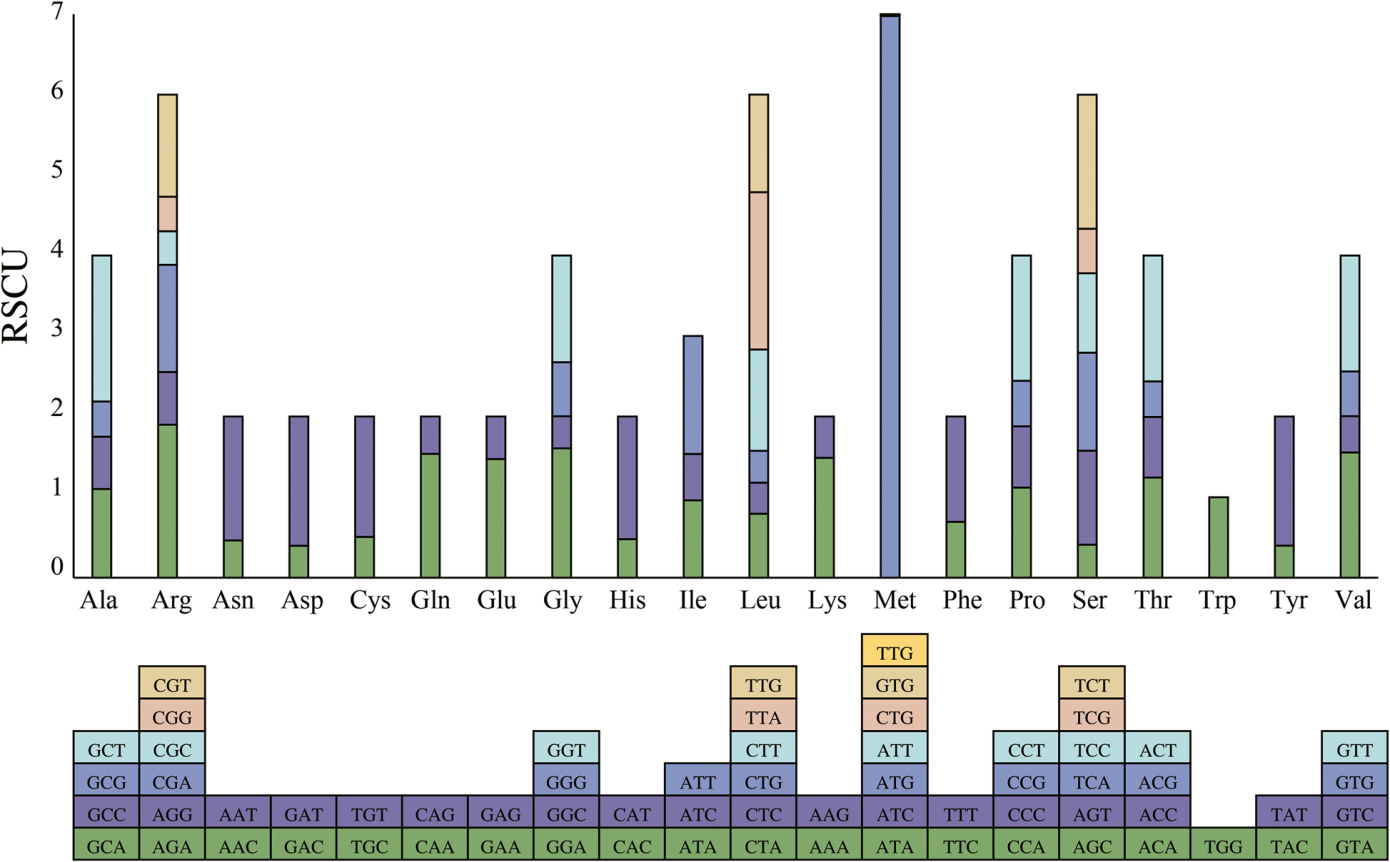
Statistical diagram of RSCU. The bottom square represents all the codons encoding each amino acid, and the height of the upper column represents the sum of the RSCU values for all codons

To assess relative synonymous codon usage (RSCU) in the coding sequences of *P. cistena* cpDNA, we analyzed 26,526 codons. The analysis revealed that AUU-I with 1117 occurrences, AAA-K with 1064 occurrences, GAA-E with 1029 occurrences, and AAU-N with 998 occurrences are the four most frequently used codons, accounting for 4.21%, 4.01%, 3.88%, and 3.76% of all codons, respectively (Table S2 and Fig. 2). Similar to the previous findings in other studies, codons ending with A or T exhibit RSCU values greater than 1, while codons ending with C or G have RSCU values less than 1 [22–24].

### Analysis of repeat sequence and simple sequence repeats (SSRs)

In the cpDNA of *P. cistena*, a comprehensive analysis revealed the presence of 49 long repeats, including 19 forward, 23 palindrome, 6 reverse, and 1 complement repeats (Fig. S1). The length of these repeat sequences ranges mainly from 30 to 26,436 bp. Furthermore, these long repeats are distributed across different regions, with 27 in the LSC region, 23 in the IRs, and 7 in the SSC region.

**Fig. 3:**
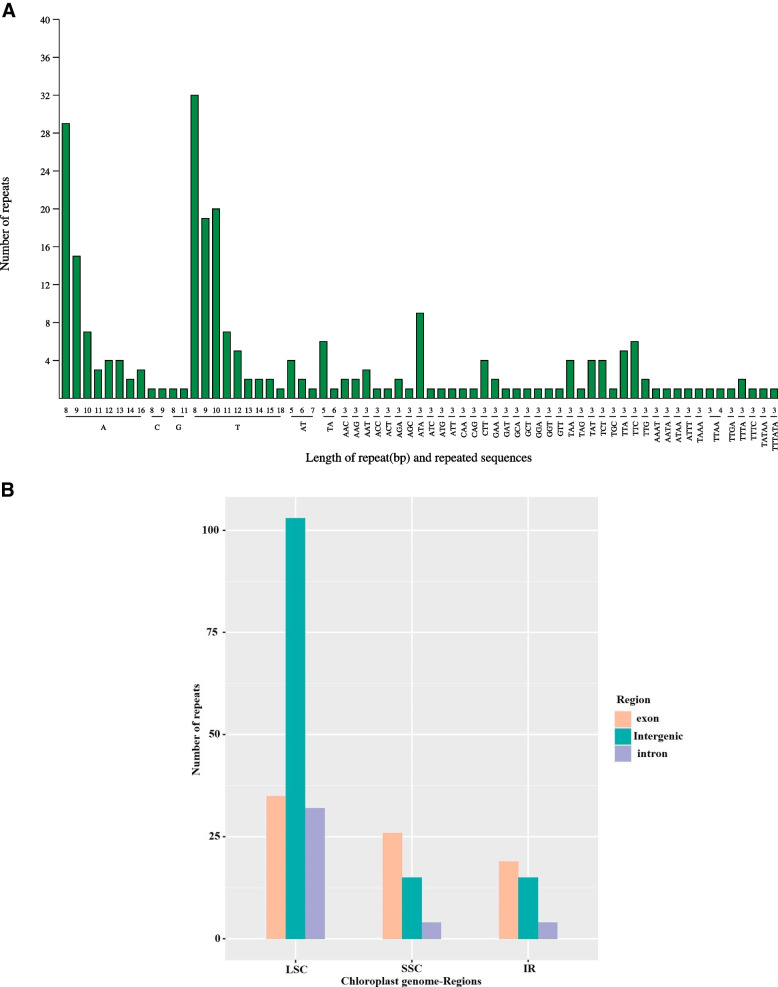
Analysis of simple sequence repeats (SSRs) in *Prunus Cistena* CP genomes. **A** Length of repeat and repeated sequences. The abscissa represents the SSR repeating unit, and the ordinate represents the number of repeating units. **B** The distribution region of SSRs

We detected 253 simple sequence repeats (SSRs) in the cpDNA. These SSRs consist of 161 mononucleotide repeats, 14 dinucleotide repeats, 65 trinucleotide repeats, 11 tetranucleotide repeats, one pentanucleotide repeat, and one hexanucleotide repeat (Fig. 3A). The mononucleotide SSRs are the most abundant, accounting for 63.63% of all SSRs, followed by trinucleotide SSRs, which account for 25.69% of the SSRs. Among these SSRs, 170 SSRs are in the LSC regions, 45 in the SSC regions, and 38 in the IR regions (Fig. 3B). Further analysis of SSR distribution in different genomic regions revealed 35 SSRs in exons, 32 in introns, and 103 in the intergenic regions of the LSC regions. In the SSC regions, 26 SSRs are in exons, 4 in introns, and 15 in the intergenic regions. Similarly, in the IR regions, 19 SSRs are in exons, 4 in introns, and 15 SSRs in the intergenic regions. The significant variability in the number of SSRs in *P. cistena* cpDNA provides valuable information for molecular marker studies and plant breeding.

### Adaptive evaluation analysis

The non-synonymous (Ka) to synonymous (Ks) ratio (Ka/Ks) was used to evaluate the degree of selection constraint on each gene and estimate the selective pressure of PCGs. Ka/Ks > 1 indicates positive selection, Ka/Ks = 1 indicates neutral selection, and Ka/Ks < 1 indicates purification selection [25].

**Fig. 4:**
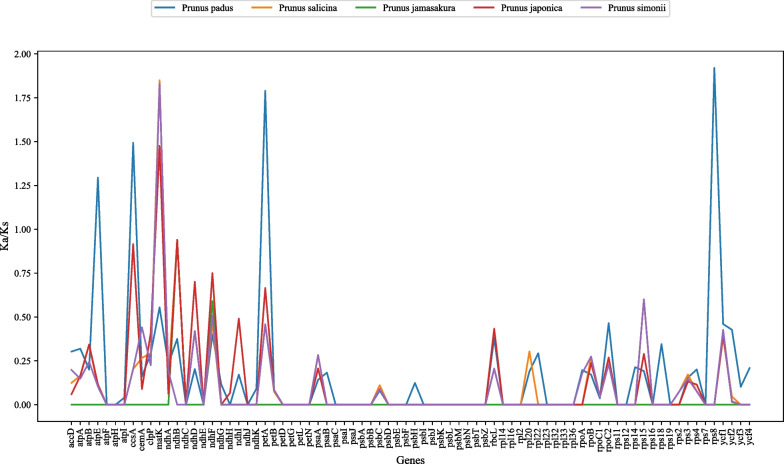
The Ka/Ks values of 78 shared genes between *P. Cistena* and other species

We used the KaKs Calculator to calculate the Ka/Ks ratios of 78 shared PCGs in *P. cistena* and five other *Prunus* species (Table S3). The results revealed a range of Ka/Ks values between *P. cistena* and *Prunus* species, spanning from 0 (*ndhC*) to 1.84989 (*matK*). Specifically, *atpE*, *ccsA*, *petA*, and *rps8* genes exhibit Ka/Ks values greater than 1 between *P. cistena* and *P. padus*, indicating positive selection effects in these genes. Moreover, *matK* gene has undergone positive selection within *P. cistena*, *P. salicina*, *P. japonica*, and *P. simonii*. On the other hand, the Ka/Ks values of the remaining genes are < 1, indicating strong purification selection pressure in the genus *Prunus* (Fig. 4).

### Phylogenetic analysis

**Fig. 5:**
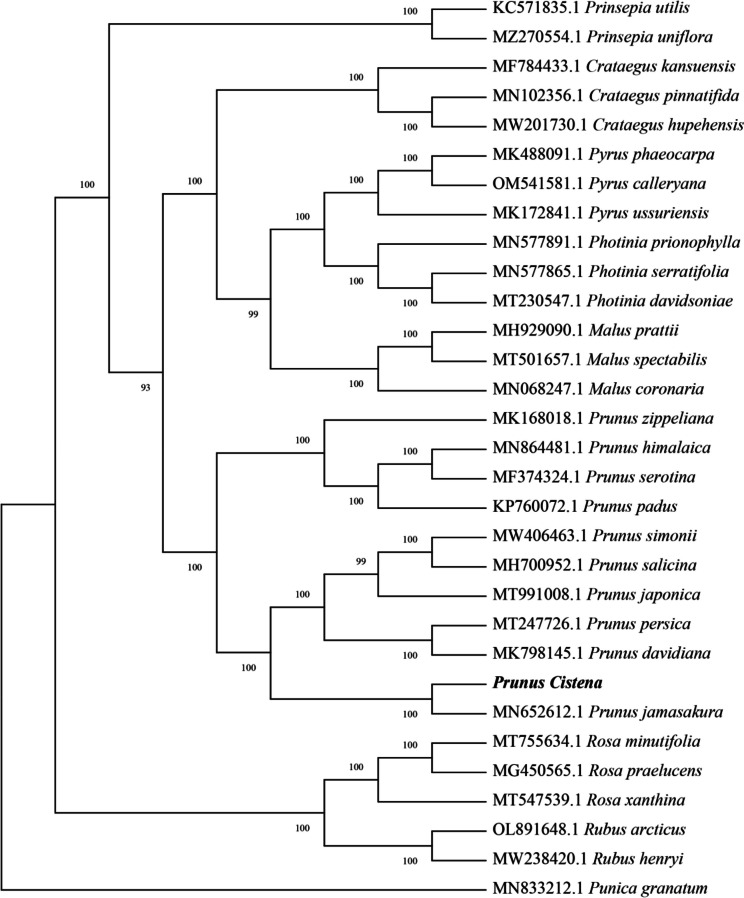
Phylogenetic tree of 31 complete cpDNAs constructed using the maximum likelihood. Bootstrap values are shown near each node

The cpDNAs of *P. cistena* and another 29 species within the Rosaceae family were selected to explore the genetic relationship between *P. cistena* and its relatives, with *Punica granatum* as the outgroup. To construct the phylogenetic tree, multiple alignments of all 31 cpDNAs were computed using MAFFT, and a maximum likelihood (ML) tree was generated using RAxML with the GTRGAMMA model. The robustness of the tree was confirmed by high bootstrap values ranging from 93 to 100 (Fig. 5).

The phylogenetic tree, characterized by strong support for most branches, revealed three divergent clades. The first clade comprises species from genus *Prinsepia*, *Crataegus*, *Pyrus*, *Photinia*, *Malus*, and *Prunus*, and is divided into three sub-categories. The *Prinsepia utilis* and *Prinsepia uniflora* are clustered into one sub-category. The second sub-category is consisting of three *Crataegus* species (*Crataegus kansuensis*, *Crataegus pinnatifida*, *Crataegus hupehensis*), three *Pyrus* species (*Pyrus phaeocarpa*, *Pyrus calleryana*, *Pyrus ussuriensis*), three *Photinia* species (*Photinia prionophylla*, *Photinia serratifolia*, *Photinia davidsoniae*), and three *Malus* species (*Malus prattii*, *Malus spectabilis*, *Malus coronaria*)*.* The third sub-category consists mainly of species from the genus *Prunus.* The second clade comprises three *Rosa* species and two *Rubus* species, includes *Rosa minutifolia*, *Rosa praelucens*, *Rosa xanthine*, *Rubus arcticus* and *Rubus henryi.* The outgroup *P. granatum* is uniquely positioned in the third clade (Fig. 5). Notably, the *Prunus* species form a closely-knit group, and *P. cistena* is closely related to *P. jamasakura* within this cluster.

### Comparative chloroplast genomic analysis

**Fig. 6:**
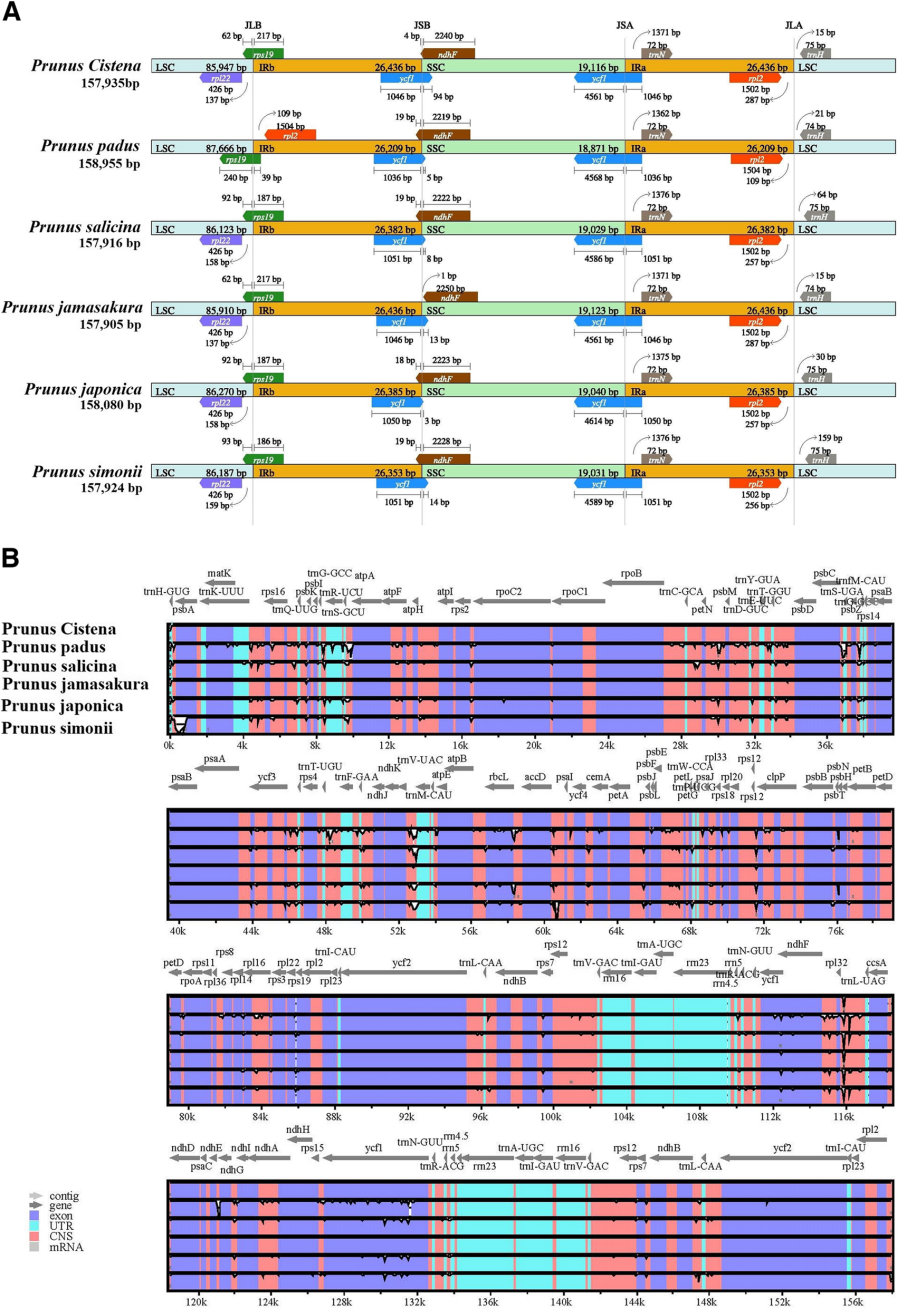
Analysis of IR boundary variation and sequence alignment of six selected *Prunus* cp genomes. **A** IR boundary variation analysis. Thin lines represent junctions for each region, and information about genes near the junctions is presented in the figure. **B** Sequence alignment analysis. The vertical scale indicates the percent identity ranging from 50 to 100%. Gray arrows and thick black lines above the alignment indicate the gene orientation

To advance our understanding of the cpDNAs in the genus *Prunus*, we conducted a further investigation to make critical comparisons of the IR/SSC and IR/LSC border positions in six selected *Prunus* species, aiming to access the degree of IR expansion or contraction among them. The observed differences in the boundary positions were apparent in the six cpDNAs, with an average length of 86,351 bp for LSC, 26,367 bp for IRa/b, and 19,035 bp for SSC regions (Fig. 6A).

Specifically, the *rpl22* gene of *P. cistena*, *P. salicina*, *P. jamasakura*, *P. japonica*, and *P. simonii* is located completely at the junctions of the LSC regions (Fig. 6A). The LSC/IRb border is situated within the *rps19* gene in the six cpDNAs. In *P. cistena and P. jamasakura,* a 217 bp fragment of the *rps19* gene is within the IRb region, while the remaining 62 bp section of the *rps19* gene is within the LSC region. In *P. padus*, a 240 bp fragment of the *rps19* is within the LSC region, while the remaining 39 bp section situates within the IRb region. The IRb/SSC boundary is inside the *ycf1* and *ndhF* genes. The length of *ycf1* fragment ranges from 1036 to 1051 bp in the IRb region, while the remaining Sect. (3 bp to 94 bp) is present in the six cpDNAs. Similarly, the *ndhF* gene has a 1 bp to 10 bp fragment located within the IRb region, while the remaining 2219 bp to 2250 bp section is within the SSC region. The *ycf1* gene spans the SSC/IRa junction, with length in the SSC region ranging from 4561 to 4614 bp. The *trnN-GUU* and *rpl2* are entirely present in the IRa region, although *rpl2* is present in the IRb region in *P. padus*. The *trnH-GUG* is entirely located in the LSC region.

To assess the degree of the genome divergence, sequence alignment and collinearity analysis were performed among the selected six species using the mVISTA and Mauve software (Fig. 6B, Fig. S2). The nucleotide sequence similarity of the six cpDNAs was extremely high, suggesting minimal variation in the cp genome of *P. cistena* compared to its ancestral species. Nonetheless, some divergence was evident in the highly conserved regions, with the coding regions and IR regions exhibiting greater conservation than the noncoding regions.

Additionally, the chloroplast genome structures of *P. cistena* and its closely related species were also analyzed using CGVIEW (Fig. S3), confirming a high similarity between the genomes. The nucleotide diversity (pi) values of the selected eight cpDNAs, calculated within the slide windows, range from 0 to 0.02398, with an average of 0.00296 (Fig. S4). Three highly variable regions, namely LSC.rps18, LSC.rbcL, and SSC.ycf1, display pi values higher than 0.01. Overall, the results indicate low pi values, suggesting that the cpDNA sequences are highly conserved at the sequence level throughout the genus *Prunus*.

## Discussion

The cp genome represents a valuable resource for molecular phylogenetic studies and serves as a productive biological and agricultural tool for fast and accurate crop recognition [26–28]. In the present study, we successfully sequenced and assembled the complete 157,935 bp long chloroplast genome of *P. cistena* (Fig. 1). The genome exhibits highly similar characteristics to other *Prunus* species in size, overall structure, gene order, and content. These findings are consistent with previous studies on the genus *Prunus*, which have shown a conserved nature of chloroplast sequences and gene arrangements [2, 4]. The GC contents in the LSC and SSC regions are significantly lower than in the IR region, primarily due to the relatively high GC content in rRNA and tRNA genes, which occupy more space than the PCGs in the IR regions [20, 29–31].

It is well known that introns regulate the expression of some genes and play an important role in alternative splicing [20, 32]. Our study observed the presence of two introns in *ycf3* and *clpP* in the *P. cistena* chloroplast genomes, consistent with the research findings in other plant species [4, 32–34]. The *ycf3* gene, known to be involved in photosynthesis [32, 35], holds potential for further investigation in *Prunus* chloroplast research. The *rps12* gene locates at the 5' end of the LSC region, with its duplicated 3' ends in the IRs regions, indicating a trans-spliced gene phenomenon [23, 27, 36]. The *ycf1* gene plays a significant role in the chloroplast genome and has been reported as a prominent pseudogene in plants, leading to incomplete gene duplication within the IRs [32, 37]. In *P. cistena*, the *ycf1* gene is repeated twice in the chloroplast genome, a feature shared with other *Prunus* plants [23, 38]. It has been testified that the introns play a significant role in regulating gene expression [20, 32, 39], which potential implication for gene expression in different spatiotemporal contexts need to be further explored.

Repeat sequences can enhance genetic diversity within species and influence cpDNA rearrangements [23, 40]. In general, cpSSRs are valuable genetic tools for population genetics and evolution studies due to their codominant nature, high polymorphism, and low substitution rate [41, 42]. The identified 253 SSRs, including six types of SSRs in the chloroplast genome of *P. cisterna*, can serve as valuable genetic markers for identifying related species. Mononucleotide SSRs are the most abundant, consistent with other *Prunus* plants [4, 34]. These SSRs can also be employed in developing lineage-specific cpSSR markers [12, 18].

Ka/Ks, which measures nucleotide substitutions, is used to quantify genomic evolution and indicates selection pressures on genes [25, 43]. In our study, the Ka/Ks value of *P. cistena* cp genome, when compared to five closely related species of genus *Prunus*, reveals an average value below one, indicating clear purifying selection on the genes. Most PCGs among the 6 *Prunus* chloroplast genomes display low rates of evolution (Ka/Ks < 1), consistent with findings in other high plant chloroplast genomes [44, 45]. However, *atpE*, *ccsA*, *petA*, *rps8*, and *matK* genes exhibit Ka/Ks values greater than one, suggesting that these genes have undergone significant positive selection in *Prunus* species, a finding supported by previous research [45, 46].

Molecular and morphological evidence has suggested that the genus *Prunus* has a complex evolutionary history [4]. Based on the complete chloroplast genome sequences of 30 species in the Rosaceae family, we performed phylogenetic analyses to determine the relationship of *P. cistena* with other speices. The results consistently indicate that *Prunus* species cluster together, with *P. cistena* showing a close evolutionary relationship with *P. jamasakura*. This finding further confirms that *P. jamasakura* is the progenitor of *P. cistena*. The phylogenetic insights provided by this study form a valuable basis for future phylogenetic analyses of species within the family Rosaceae.

Regarding the chloroplast genome structure, the IR region is typically the most conserved region. The modification of cpDNA size can result from important evolutionary events, such as expansion and shrinkage of the IR regions, leading to fluxes in the LSC/IR junctions, initiation of pseudogenes, and gene duplications [4, 47]. The LSC/SSC and IR region junctions are known to be highly conserved among angiosperm cpDNAs [18, 48]. In the cpDNAs of six *Prunus* species examined in this study, *rps19* gene is located at the boundary of LSC/IRb, with the length in the IRb regions longer than that in the LSC region, except for *P. padus*. The junction of the LSC/IRb boundary is between *rpl2* and *rps19* in *P. padus*, while in the other species, it is between *rpl22* and *rps19*, which has also been reported in other plants [49, 50]. The *ycf1* gene is in the IRa/SSC and IRb/SSC regions, with 4,561 bp to 4,614 bp in the IRa region and 1,036 bp to 1,051 bp in IRb region, and the pseudogenized overlapping segments of *ycf1* are present at the junction of JSB (the connection between SSC area and IRb area) in all six selected *Prunus* species, in line with earlier reports [23, 32].

The nucleotide sequence similarity among the six *Prunus* cpDNAs was extremely high, with the coding and IR regions exhibiting higher conservation compared to the noncoding regions. This indicates a highly conserved nature of the cpDNA sequences throughout the genus *Prunus* [4, 51]. The *rps18* gene in the LSC region shows the highest Pi value in the chloroplast genomes of *P. cistena*, making it a potential candidate for phylogenetic analysis and population genetic study in *Prunus* species. These findings contribute to our understanding of the evolutionary patterns within the Rosaceae family.

## Conclusion

Employing the Illumina Hiseq platform, we successfully sequenced and assembled the complete chloroplast genome of *P. cistena* and compared it with the cp genomes of other *Prunus* species. The results show that the chloroplast sequences and gene arrangements in *P. cistena* are highly conserved, with similar size, overall structure, gene order, and content compared to other *Prunus* species. The *atpE*, *ccsA*, *petA*, *rps8,* and *matK* genes have undergone significant positive selection in *Prunus* species, suggesting their potential roles in the evolutionary dynamics of this genus. The phylogenetic relationships among 30 Rosaceae species strongly support the known classification of *P. cistena*. The analysis reveals that both the coding and IR regions of the chloroplast genome are more conserved than the noncoding region. These findings highlight the functional importance and evolutionary stability of these regions in *Prunus* species. Moreover, the high conservation of cpDNA sequences across the genus *Prunus* reinforces the notion of a shared evolutionary history among these species. The hotspot genes, *ycf1* and *rps18*, were identified, and they contain valuable information for species identification and phylogenetic reconstruction of *Prunus*. These genes offer promising avenues for further research to deepen our understanding of the evolutionary process within the genus *Prunus*.

## Materials and methods

### Plant material

The *P. cistena* was cultivated in the germplasm resource nursery of the Shandong Institute of Pomology, Taian City, Shandong Province, China. It is a hybrid of *P. cerasifera* and *P. jamasakura*, displaying purplish red or dark purplish red leaves. The flower is solitary with pale pink colors, and flowering from April to May.

### DNA extraction and sequencing

To preserve the DNA integrity, the fresh leaves of *P. cistena* was collected and immediately frozen in liquid nitrogen and stored at -80℃. Genomic DNA was extracted from the fresh leaves using the plant genomic DNA Kit (DP180123) from China Tiangen Biotechnology (Beijing) Co., Ltd. Once the quality of the isolated DNA was confirmed, it was fragmented into small pieces using sonication. Subsequently, the fragmented DNA was subjected to fragment purification, end repair, and the addition of an A-tail at the 3' end. Sequencing adaptors were then ligated to the prepared DNA fragments. Suitable fragments were identified through agarose gel electrophoresis and were purified for use as templates in the subsequent PCR amplification to create the final DNA library. The qualified libraries were subjected to paired-end (PE) sequencing on the Illumina NovaSeq 6000 platform by Nanjing Genepioneer Biotechnologies, with a read length of 150 bp.

### Chloroplast genome assembly and annotation

The raw reads obtained from the sequencing were filtered using Fastp v0.20.0 software (https://github.com/OpenGene/fastp)to remove adaptors and reads with an average quality below Q5 and N number greater than 5. The resulting high-quality clean data were assembled using SPAdes v3.10.1, SSPACE v2.0, and Gapfiller v2.1.1 software [52]. The CP genome of *P. simonii* (NCBI accession number MW406463.1) was used for quality control after assembly.

To ensure the accurate annotation of the the chloroplast genome, two methods were employed. First, the CDS, rRNA, and tRNA genes were aligned using prodigal v2.6.3 (https://www.github.com/hyattpd/Prodigal), HMMER v3.1b2 (http://www.hmmer.org/), and aragorn v1.2.38 (http://130.235.244.92/ARAGORN/), respectively. Second, gene sequences from closely related species, available on NCBI, were extracted and aligned with the assembled sequences using blast v2.6 (https://blast.ncbi.nlm.nih.gov/Blast.cgi). The annotation results from both methods were manually checked, and any differential genes and erroneous or redundant annotations were removed. Additionally, multi-exon boundaries were determined to obtain the final annotation of the chloroplast genome. The chloroplast genome maps were visualized using OGDRAW software [53].

### Identification of repeat sequences and cpSSR

Interspersed repetitive sequences were identified using vmatch v2.3.0 (http://www.vmatch.de/) software in combination with a Perl script. The repeat sequences encompassed forward, reverse, complement, and palindromic repeats, with the following parameters settings: minimal repeat size of 30 bp and a hamming distance of 3.

Simple sequence repeats of chloroplast genomes (cpSSR) were analyzed using MISA v1.0 software (MIcroSAtellite identification tool, http://pgrc.ipk-gatersleben.de/misa/misa.html). The parameter settings were as followings: a minimum number of eight repeated motifs for mononucleotide, five repeated motifs for dinucleotide, four repeated motifs for trinucleotide, and three repeated motifs for tetra-, penta-, and hexanucleotide repeats.

### Codon usage, selectivepressure and nucleotide diversity

Relative Synonymous Codon Usage (RSCU) is a valuable metric used to analyze genetic and evolutionary processes. It represents the ratio of the actual number of synonymous codons utilized to translate a specific amino acid to the expected number [19]. The CodonW v1.4.2 software was used to calculate RSCU and analyze codon preference in chloroplast genomes.

For indel identification, the mafft v7.310 software (https://mafft.cbrc.jp/alignment/software/) was utilized. To assess selective pressures on genes, Ka/Ks values (representation of selective pressure) were calculated using KaKs_Calculator v2.0 software (https://sourceforge.net/projects/kakscalculator2/). A Ka/Ks ratio greater than one suggests a positive selection effect, while a ratio less than one indicates purification selection.

### Phylogenetic analysis

To reveal the evolutionary relationship among the six *Prunus* species within the Rosaceae family, a comprehensive dataset comprising 29 complete chloroplast genomes of the Rosaceae family was gathered from GenBank. For phylogenetic analysis, *Punica granatum* was selected as the outer group. The sequences were aligned using MAFFT v7.427 (-auto mode), and the maximum likelihood (ML) phylogenetic tree was constructed using RAxML v8.2.10 software with GTRGAMMA model and 1000 bootstrap replicates.

### Genomic comparison with related species

Perl-IRscope (https://github.com/xul962464/perl-IRscope) was used to analyze and visualize the borders between the LSC/IRs and SSC/IRs regions in the cp genomes of six species, including *P. cistena, P. padus*, *P. salicina*, *P. jamasakura*, *P. japonica,* and *P. simonii*. The homology and collinearity of chloroplast sequences were analyzed using Mauve (http://darlinglab.org/mauve) and mVISTA software (http://genome.lbl.gov/vista/index.shtml). CGVIEWsoftware (http://stothard.afns.ualberta.ca/cgview_server/) was used to compare the complete *P. cistena* cp genome structure to that of five related species. DnaSP v5.0 software with default settings was used to calculate each gene's nucleotide diversity (Pi) value.

### Supplementary Information

**Additional file 1:** **Fig. S1. **Statistical plot of interspersed repeats sequences.The abscissa is the length of the interspersed repeats, and the ordinate is the number of interspersed repeats. F stands for forward repeat, P for palindromic repeat, R for reverse repeat, and C for complementary repeat. **Fig. S2.** Collinearity analysis of chloroplast genome sequence. The long squares represent the similarity between genomes, and the lines between the long squares represent a collinear relationship. Short squares represent gene positions for each genome. Where white represents CDS, green represents tRNA, and red represents rRNA. **Fig. S3. **Comparative analysis of chloroplast structure of *P. cistena* and proximal species. The two outermost circles describe the length and direction of genes in the genome; the circles inside represent similar results compared with other reference genomes. The black circles represent GC content, Green represents GC-skew+ and purple represents GC-skew-. **Fig. S4. **Comparative analysis of the gene nucleotide variability (pi) values of six *Prunus *species.The X-axis and Y-axis show the genes and the pi values, respectively. **Table S1. **Genes with introns in the* Prunus cistena* CP genomes. **Table S2. **RSCU usage of *Prunus cistena* CP genome. **Table S3. **The Ka/Ks value of* P. cistena *and five other *Prunus *species.

## Data Availability

The chloroplast genome sequences have been deposited in GenBank under the accession numbers: ON585706. Row data are available at SRA, under the accession number: SRR19352552.
