# Supplementary material for: Complete chloroplast genome sequences of the ornamental plant Prunus cistena and comparative and phylogenetic analyses with its closely related species
Source: BMC Genomics. 2023 Dec 5;24:739. doi: 10.1186/s12864-023-09838-9 (PMC10696862; doi:10.1186/s12864-023-09838-9)
Supplement: Supplementary file 1 — Additional file 1: Fig. S1. Statistical plot of interspersed repeats sequences.The abscissa is the length of the interspersed repeats, and the ordinate is the number of interspersed repeats. F stands for forward repeat, P for palindromic repeat, R for reverse repeat, and C for complementary repeat. Fig. S2. Collinearity analysis of chloroplast genome sequence. The long squares represent the similarity between genomes, and the lines between the long squares represent a collinear relationship. Short squares represent gene positions for each genome. Where white represents CDS, green represents tRNA, and red represents rRNA. Fig. S3. Comparative analysis of chloroplast structure of P. cistena and proximal species. The two outermost circles describe the length and direction of genes in the genome; the circles inside represent similar results compared with other reference genomes. The black circles represent GC content, Green represents GC-skew+ and purple represents GC-skew-. Fig. S4. Comparative analysis of the gene nucleotide variability (pi) values of six Prunus species.The X-axis and Y-axis show the genes and the pi values, respectively. Table S1. Genes with introns in the Prunus cistena CP genomes. Table S2. RSCU usage of Prunus cistena CP genome. Table S3. The Ka/Ks value of P. cistena and five other Prunus species. [file 12864_2023_9838_MOESM1_ESM.docx]

**Supporting information**

**
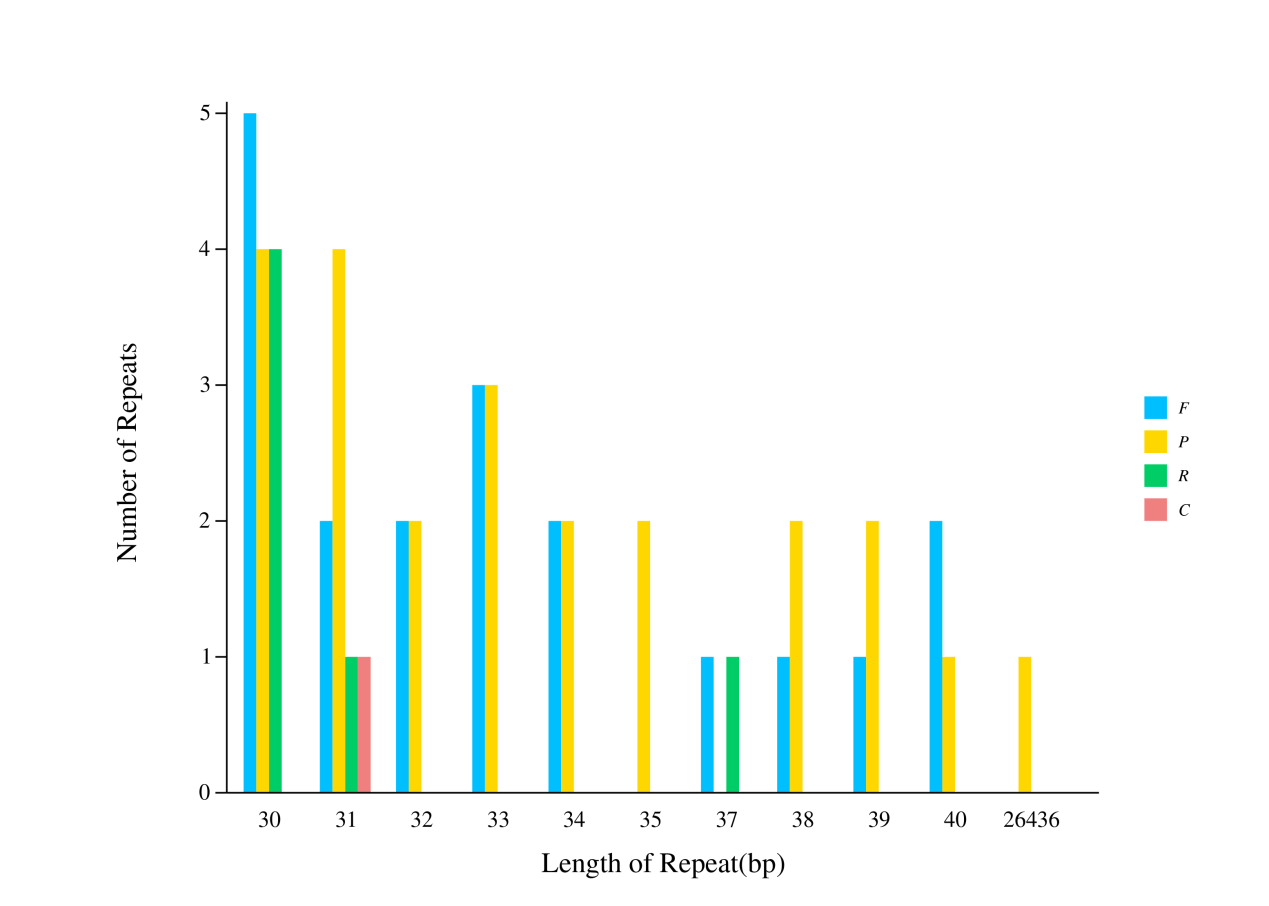
**

**Fig.S1** Statistical plot of interspersed repeats sequences. The abscissa is the length of the interspersed repeats, and the ordinate is the number of interspersed repeats. F stands for forward repeat, P for palindromic repeat, R for reverse repeat, and C for complementary repeat.

**
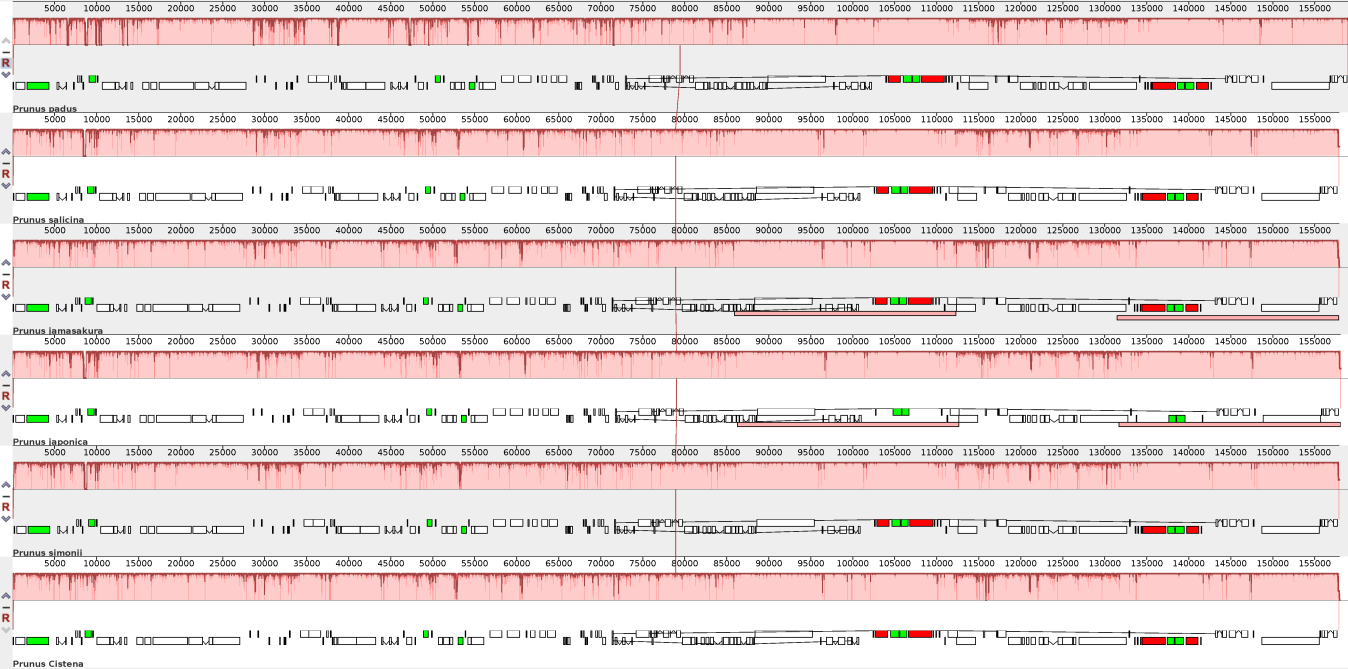
**

**Fig. S2** Collinearity analysis of chloroplast genome sequence. The long squares represent the similarity between genomes, and the lines between the long squares represent a collinear relationship. Short squares represent gene positions for each genome. Where white represents CDS, green represents tRNA, and red represents rRNA.

**
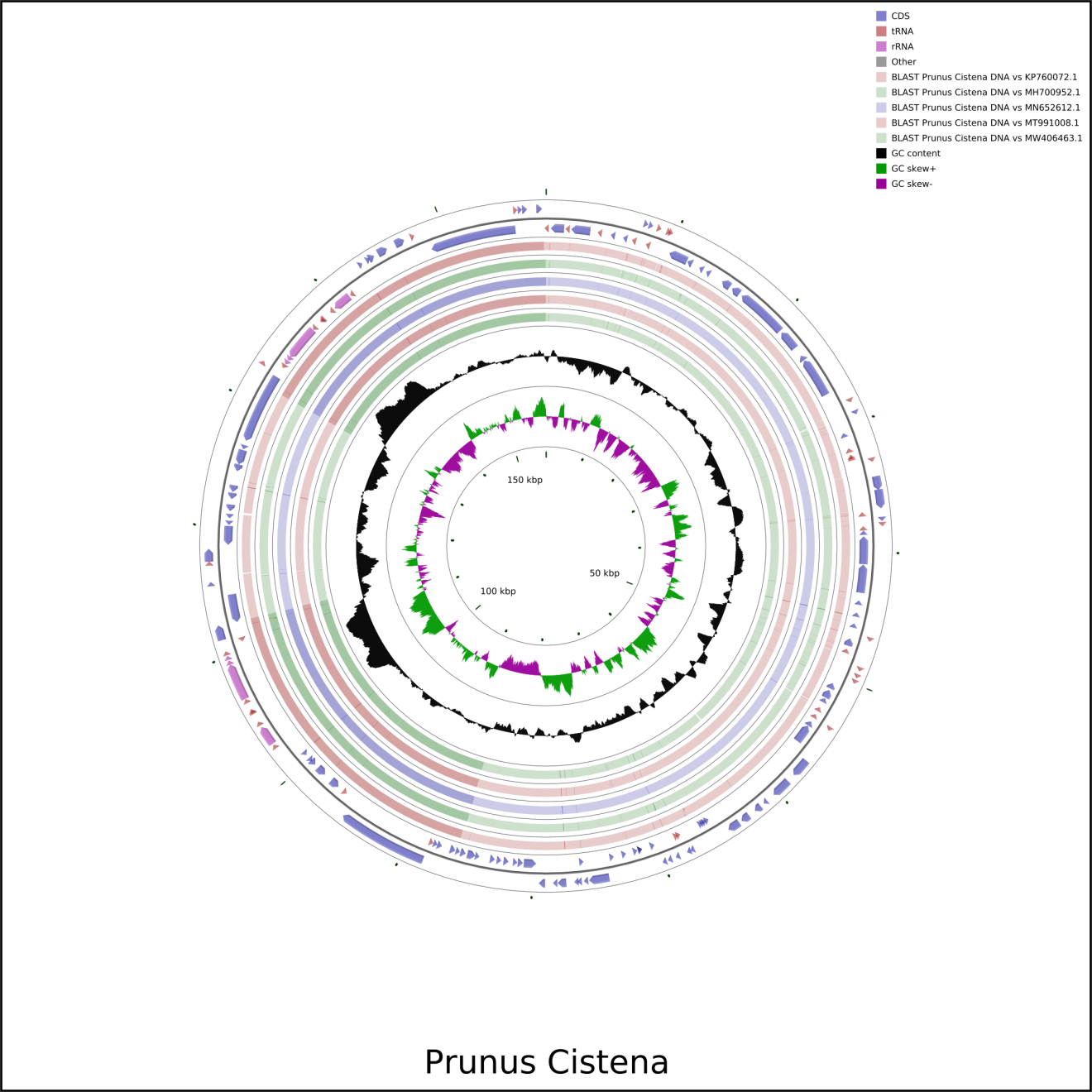
**

**Fig. S3** Comparative analysis of chloroplast structure of *P. cistena* and proximal species. The two outermost circles describe the length and direction of genes in the genome; the circles inside represent similar results compared with other reference genomes. The black circles represent GC content, Green represents GC-skew+ and purple represents GC-skew-.


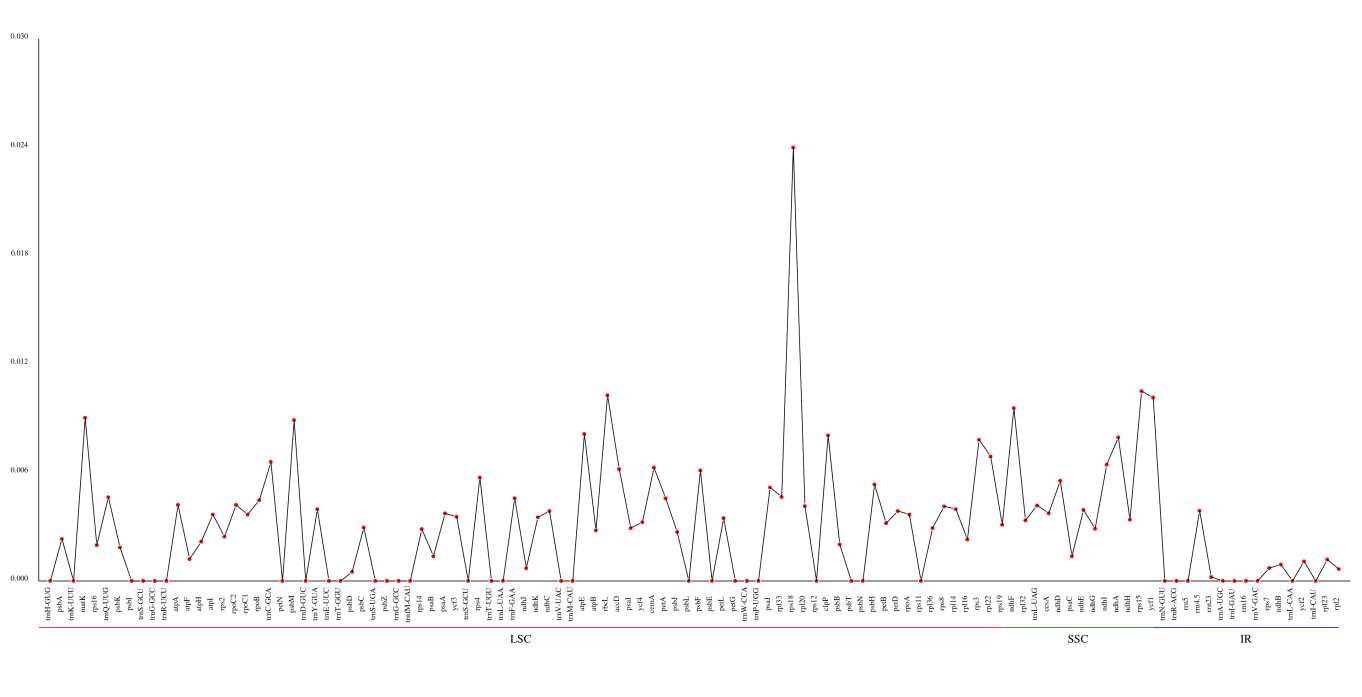


**Fig. S4** Comparative analysis of the gene nucleotide variability (pi) values of six *Prunus* species. The X-axis and Y-axis show the genes and the pi values, respectively.

**Table S1** Genes with introns in the *Prunus cistena* CP genomes

| Gene | Location | Exon I(bp) | Intron II(bp) | Exon II(bp) | Intron II(bp) | Exon III(bp) |
| --- | --- | --- | --- | --- | --- | --- |
| trnK-UUU  rps16  trnG-GCC  atpF  rpoC1  ycf3  trnL-UAA  trnV-UAC  rps12  clpP  petB  petD  rpl16  rpl2  ndhB  rps12  trnI-GAU  trnA-UGC  ndhA  trnA-UGC  trnI-GAU  ndhB  rpl2 | LSC  LSC  LSC  LSC  LSC  LSC  LSC  LSC  IRa  LSC  LSC  LSC  LSC  IRb  IRb  IRb  IRb  IRb  SSC  IRa  IRa  IRa  IRa | 37  40  23  145  434  124  37  39  114  71  6  8  9  385  775  232  42  38  553  38  42  775  385 | 2530  863  714  746  760  776  514  589  -  669  758  743  1018  683  680  -  943  807  1139  807  943  680  683 | 35  230  49  410  1618  230  50  37  232  291  642  475  399  434  758  26  35  35  539  35  35  758  434 | 712  538  814  538 | 153  26  226  114 |

**Table S2** RSCU usage of *Prunus cistena* CP genome

| AminoAcid | Symbol | Codon | No. | RSCU |
| --- | --- | --- | --- | --- |
| * | Ter | UAA | 49 | 1.7295 |
| * | Ter | UAG | 20 | 0.7059 |
| * | Ter | UGA | 16 | 0.5646 |
| A | Ala | GCA | 386 | 1.1028 |
| A | Ala | GCC | 226 | 0.6456 |
| A | Ala | GCG | 153 | 0.4372 |
| A | Ala | GCU | 635 | 1.8144 |
| C | Cys | UGC | 79 | 0.508 |
| C | Cys | UGU | 232 | 1.492 |
| D | Asp | GAC | 214 | 0.3974 |
| D | Asp | GAU | 863 | 1.6026 |
| E | Glu | GAA | 1029 | 1.4752 |
| E | Glu | GAG | 366 | 0.5248 |
| F | Phe | UUC | 525 | 0.695 |
| F | Phe | UUU | 986 | 1.305 |
| G | Gly | GGA | 718 | 1.608 |
| G | Gly | GGC | 176 | 0.394 |
| G | Gly | GGG | 300 | 0.672 |
| G | Gly | GGU | 592 | 1.326 |
| H | His | CAC | 155 | 0.4798 |
| H | His | CAU | 491 | 1.5202 |
| I | Ile | AUA | 735 | 0.9615 |
| I | Ile | AUC | 441 | 0.5769 |
| I | Ile | AUU | 1117 | 1.4613 |
| K | Lys | AAA | 1064 | 1.4902 |
| K | Lys | AAG | 364 | 0.5098 |
| L | Leu | CUA | 368 | 0.7962 |
| L | Leu | CUC | 178 | 0.3852 |
| L | Leu | CUG | 183 | 0.396 |
| L | Leu | CUU | 580 | 1.2546 |
| L | Leu | UUA | 904 | 1.9554 |
| L | Leu | UUG | 561 | 1.2132 |
| M | Met | AUA | 0 | 0 |
| M | Met | AUC | 0 | 0 |
| M | Met | AUG | 621 | 6.9776 |
| M | Met | AUU | 0 | 0 |
| M | Met | CUG | 0 | 0 |
| M | Met | GUG | 1 | 0.0112 |
| M | Met | UUG | 1 | 0.0112 |
| N | Asn | AAC | 302 | 0.4646 |
| N | Asn | AAU | 998 | 1.5354 |
| P | Pro | CCA | 305 | 1.1204 |
| P | Pro | CCC | 206 | 0.7568 |
| P | Pro | CCG | 154 | 0.5656 |
| P | Pro | CCU | 424 | 1.5572 |
| Q | Gln | CAA | 718 | 1.5392 |
| Q | Gln | CAG | 215 | 0.4608 |
| R | Arg | AGA | 504 | 1.8972 |
| R | Arg | AGG | 174 | 0.6552 |
| R | Arg | CGA | 354 | 1.3326 |
| R | Arg | CGC | 111 | 0.4176 |
| R | Arg | CGG | 114 | 0.429 |
| R | Arg | CGU | 337 | 1.2684 |
| S | Ser | AGC | 138 | 0.4104 |
| S | Ser | AGU | 393 | 1.1688 |
| S | Ser | UCA | 408 | 1.2138 |
| S | Ser | UCC | 332 | 0.9876 |
| S | Ser | UCG | 186 | 0.5532 |
| S | Ser | UCU | 560 | 1.6656 |
| T | Thr | ACA | 422 | 1.2448 |
| T | Thr | ACC | 254 | 0.7492 |
| T | Thr | ACG | 150 | 0.4424 |
| T | Thr | ACU | 530 | 1.5636 |
| V | Val | GUA | 564 | 1.5568 |
| V | Val | GUC | 162 | 0.4472 |
| V | Val | GUG | 201 | 0.5548 |
| V | Val | GUU | 522 | 1.4408 |
| W | Trp | UGG | 451 | 1 |
| Y | Tyr | UAC | 201 | 0.3988 |
| Y | Tyr | UAU | 807 | 1.6012 |

**Table S3** The Ka/Ks value of *P. cistena* and five other *Prunus* species

| Gene | Prunus padus | Prunus salicina | Prunus jamasakura | Prunus japonica | Prunus simonii |
| --- | --- | --- | --- | --- | --- |
| accD | 0.302797 | 0.124709 | NA | 0.0600698 | 0.198641 |
| atpA | 0.319272 | 0.165125 | NA | 0.165125 | 0.148213 |
| atpB | 0.199769 | 0.342784 | NA | 0.342784 | 0.244085 |
| atpE | 1.29511 | 0.103455 | NA | 0.116025 | 0.103455 |
| atpF | 0 | NA | NA | NA | NA |
| atpH | 0 | 0 | NA | 0 | 0 |
| atpI | 0.0400462 | 0 | 0 | 0 | 0 |
| ccsA | 1.49325 | 0.203017 | NA | 0.915715 | 0.203017 |
| cemA | 0.161445 | 0.266163 | 0 | 0.0893453 | 0.44111 |
| clpP | 0.290857 | 0.29436 | NA | 0.410044 | 0.224684 |
| matK | 0.555007 | 1.84989 | NA | 1.47539 | 1.83059 |
| ndhA | 0.231906 | 0.144713 | NA | 0.0621134 | 0.191129 |
| ndhB | 0.375166 | 0.939514 | 0.939514 | 0.939514 | NA |
| ndhC | 0 | 0 | NA | 0 | 0 |
| ndhD | 0.202708 | 0.4196 | 0 | 0.701062 | 0.4196 |
| ndhE | 0 | 0 | NA | 0 | 0 |
| ndhF | 0.401157 | 0.466581 | 0.590539 | 0.749918 | 0.508945 |
| ndhG | 0.11589 | 0 | 0 | 0 | 0 |
| ndhH | 0 | 0 | 0 | 0.0667897 | 0 |
| ndhI | 0.171384 | NA | NA | 0.490755 | NA |
| ndhJ | NA | NA | NA | NA | NA |
| ndhK | 0.091264 | 0 | NA | 0 | 0 |
| petA | 1.78933 | 0.457997 | NA | 0.665176 | 0.457997 |
| petB | 0.0842411 | 0.0739601 | NA | 0.0837446 | 0.0842411 |
| petD | 0 | 0 | NA | 0 | 0 |
| petG | NA | NA | NA | NA | NA |
| petL | 0 | NA | NA | NA | NA |
| petN | NA | NA | NA | NA | NA |
| psaA | 0.141642 | 0.282832 | NA | 0.206514 | 0.282832 |
| psaB | 0.182955 | 0 | 0 | 0 | 0 |
| psaC | 0 | NA | NA | NA | NA |
| psaI | 0 | NA | NA | NA | NA |
| psaJ | NA | NA | NA | NA | 0 |
| psbA | 0 | 0 | NA | 0 | 0 |
| psbB | 0 | 0 | NA | 0 | 0 |
| psbC | 0.0804748 | 0.111073 | NA | 0.089023 | 0.089023 |
| psbD | 0 | 0 | NA | 0 | 0 |
| psbE | NA | NA | NA | NA | NA |
| psbF | 0 | 0 | NA | 0 | 0 |
| psbH | 0.124115 | NA | NA | NA | NA |
| psbI | NA | NA | NA | NA | NA |
| psbJ | 0 | NA | NA | NA | NA |
| psbK | 0 | NA | NA | NA | NA |
| psbL | NA | NA | NA | NA | NA |
| psbM | 0 | NA | NA | NA | NA |
| psbN | NA | NA | NA | NA | NA |
| psbT | NA | NA | NA | NA | NA |
| psbZ | NA | NA | NA | NA | NA |
| rbcL | 0.38152 | 0.205689 | NA | 0.433165 | 0.205689 |
| rpl14 | NA | 0 | NA | 0 | 0 |
| rpl16 | 0 | 0 | NA | 0 | 0 |
| rpl2 | NA | 0 | NA | NA | 0 |
| rpl20 | 0.189359 | 0.303322 | NA | 0 | 0 |
| rpl22 | 0.293394 | 0 | NA | 0 | 0 |
| rpl23 | NA | NA | NA | NA | NA |
| rpl32 | 0 | 0 | NA | 0 | 0 |
| rpl33 | 0 | NA | NA | NA | NA |
| rpl36 | 0 | NA | NA | NA | NA |
| rpoA | 0.197538 | 0 | NA | 0 | 0.179607 |
| rpoB | 0.17167 | 0.274769 | NA | 0.238656 | 0.274223 |
| rpoC1 | 0.0362463 | 0.0467047 | 0 | 0.0467047 | 0.0410669 |
| rpoC2 | 0.464889 | 0.266261 | NA | 0.268884 | 0.230605 |
| rps11 | NA | NA | NA | NA | NA |
| rps12 | NA | NA | NA | NA | NA |
| rps14 | 0.214016 | 0 | NA | 0 | 0 |
| rps15 | 0.191715 | 0.600257 | NA | 0.2894 | 0.600257 |
| rps16 | 0 | 0 | NA | 0 | 0 |
| rps18 | 0.345542 | NA | NA | NA | NA |
| rps19 | 0 | 0 | NA | 0 | 0 |
| rps2 | 0 | 0.0738257 | 0 | 0 | 0.0738257 |
| rps3 | 0.154401 | 0.17262 | NA | 0.132098 | 0.148091 |
| rps4 | 0.201132 | 0.0762052 | NA | 0.114768 | 0.0762052 |
| rps7 | NA | NA | NA | NA | NA |
| rps8 | 1.9199 | NA | NA | NA | NA |
| ycf1 | 0.459769 | 0.383244 | NA | 0.401974 | 0.427172 |
| ycf2 | 0.427092 | 0.0463348 | 0 | 0.0182562 | 0.0154382 |
| ycf3 | 0.101913 | 0 | 0 | 0 | 0 |
| ycf4 | 0.208711 | 0 | NA | 0 | 0 |
